# Preventing healthcare-associated MRSA bacteremia: getting to the root of the problem

**DOI:** 10.1017/ash.2023.518

**Published:** 2023-12-22

**Authors:** Michael A. Borg, David Suda, Ermira Tartari, Claire Farrugia, Deborah Xuereb, Monique Borg Inguanez

**Affiliations:** 1 University of Malta, Msida, Malta; 2 Department of Infection Prevention & Control, Mater Dei Hospital, Msida, Malta

## Abstract

**Introduction:**

Bloodstream infections caused by methicillin-resistant *Staphylococcus aureus* (MRSA) remain a major challenge in most countries worldwide.

**Setting:**

We describe a quasi-experimental sequential intervention at Mater Dei Hospital, Malta, to reduce hyper-prevalence of healthcare-associated MRSA bacteremia (HA-MRSA-B).

**Interventions:**

The hospital initiated a hand hygiene (HH) campaign in 2008 to improve alcohol hand rub (AHR) use. In 2011, this was followed by root cause analysis (RCA) of all HA-MRSA-B cases and finally universal MRSA admission screening in 2014. Change-point analysis was used to evaluate the impact of the interventions.

**Results:**

The effect of the HH campaign became evident when AHR consumption reached 40 L/1000 occupied bed days (BD). RCAs identified intravascular devices as the likely risk factor in 83% of all HA-MRSA-B; specifically non-tunneled double-lumen hemodialysis catheters (36%), peripheral venous cannulas (25%), and central venous catheters (22%). Interventions to improve their management resulted in the greatest reduction of HA-MRSA-B rates. They were informed by the RCA findings and targeted behavior change through education, motivation, and system change. Universal MRSA admission screening provided the final decline in incidence. Each intervention affected HA-MRSA-B rates after a lag period of approximately 18–24 months. Overall, HA-MRSA-B incidence decreased from 1.72 cases/10000BD in 2008 to 0.18/10000BD in 2019; a reduction of almost 90%. Intravenous device interventions were also associated with a reduction of methicillin-sensitive *Staphylococcus aureus* (MSSA) bacteremia rates.

**Conclusions:**

Significant improvement in HA-MRSA-B is possible, even in highly endemic regions. It requires well-planned behavior change interventions which are compatible with local context and culture.

## Introduction

Bloodstream infections (BSIs) caused by *Staphylococcus aureus* exceed 50 cases/100,000 population, even in high-resource countries, with a mortality rate approximating 20%–30%.^1^ Antimicrobial resistance (AMR) adds an even greater impact through higher 30-day and in-hospital mortality rates.^2^ The burden of BSIs caused by methicillin-resistant *Staphylococcus aureus* (MRSA) is not just restricted to patient outcomes.^3^ MRSA BSIs account for more than 250,000 extra bed days (BDs) in countries of the European Union (EU) and European Economic Area (EEA).^4^ Several regions have reported an apparent reduction in MRSA proportions from blood culture *S. aureus* isolates.^5^ However, despite a significant reduction in resistance proportions, Cassini *et al*. estimated that the incidence of MRSA BSIs in the EU/EEA actually increased by 1.28 times between 2007 and 2015.^3^ MRSA bacteremia therefore remains an important medical challenge which demands effective efforts at prevention and control.

## Setting

Mater Dei Hospital (MDH) is the sole tertiary care hospital in Malta, a Mediterranean island with a population of approximately 500,000. This 1000-bed facility provides various specialist services, including intensive care, transplantation, and complex surgery. Not surprisingly, it is the main contributor to the country’s AMR epidemiology. For the best part of the 1990s and 2000s, infections caused by MRSA were hyper-prevalent. In 2008, Davey *et al* identified Malta as having the second highest incidence of MRSA bacteremia in the EU/EAA.^6^ We outline sequential interventions to address this challenge in the subsequent decade. For the purpose of this publication, a case of healthcare-associated MRSA bacteremia (HA-MRSA-B) was defined as an MRSA isolate grown from a blood culture taken 48 hours or later after admission to hospital or preceded by an intervention in the previous 30 days (such as surgery or hemodialysis).

## Interventions

### Hand hygiene campaign

Surveillance data on MRSA bacteremia, following participation in EU/EEA surveillance networks, elicited a previously absent sense of urgency to address the problem. This coincided with the launch of the World Health Organization’s “Clean Care is Safer Care” Global Patient Safety initiative, promoting improved hand hygiene (HH) through increased alcohol hand rub (AHR) use at critical moments of patient contact.^7^ A HH campaign was launched in 2008 and has been described elsewhere.^8^ It focused heavily on extensive audits of HH compliance and monitoring of AHR use, which we have previously shown to correlate well with assessment of HH performance through visual observations at MDH.^9^

### Root cause analysis

At around this time, the successful efforts of the United Kingdom to address its hitherto high MRSA prevalence were being highlighted.^10^ A cornerstone of this initiative was the requirement to perform a root cause analysis (RCA) for each case of HA-MRSA-B, conducted by the patient’s clinical team. The protocol required early gathering of data to find out what happened, generating an action plan to address the key issues identified and the implementation and monitoring of an action plan which would feed learning into an organization’s governance to help reduce the chances of a repetition.^11^ In 2010, an attempt was made to leverage positive deviance from this experience and implement a similar approach in MDH. However, initial experiments to replicate the UK administrative model were unsuccessful, primarily due to lack of ownership by front-line professionals. RCAs only materialized when the hospital’s Infection Prevention and Control (IPC) team took over their full management. IPC performs the preliminary review of the case and invites all the key stakeholders involved in the patient’s care to a meeting, led by the IPC Lead. The clinical team provides the background which is then followed by a discussion, asking the “five why’s?” to identify possible factors.^12^ Every effort is made to ensure that the meeting takes place in a safe, non-punitive environment and that everyone can provide their input, keeping the focus on the event and related processes. At the end of the meeting, based on RCA conclusions, action items are agreed and minuted. The minutes and corrective actions are sent to all meeting participants and copied to senior management. The IPC team reviews implementation of agreed corrective actions and raises any identified lack of progress with senior hospital management.

An assessment of the RCAs held in 2011, the first full year of the initiative, showed that the absolute majority of cases were attributed to intravenous (IV) devices. Most were linked to renal dialysis (36%) where almost all patients had had a non-tunneled double-lumen hemodialysis catheter (NTDLHC) at the time of the HA-MRSA-B. A further 25% of HA-MRSA-B were linked to peripheral vascular cannulas (PVC) and 22% to central venous catheters (CVC). In practically all of these RCAs, clear practice gaps were identified and evidence-based corrective actions instituted hospital-wide (Table 1).

**Table 1.** Main factors identified from root cause analysis (RCA) reviews held in 2011 and corrective actions implemented.

Peripheral vascular cannulae (25%)		Central venous catheters (22%)		Renal dialysis catheters (36%)	
Gap identified	Corrective action	Gap identified	Corrective action	Gap identified	Corrective action
Inappropriate PVC insertion techniques	Education for doctors and nurses of PVC insertion and maintenance, including mandatory induction training	Various deficiencies in proper attire, skin disinfection, and draping for CVC insertion	Development of insertion policy and documentation followed by training for intensivists	Long time lag between start of dialysis and having a functional A-V fistula	Any patient diagnosed at CKD stage 5 needed to have A-V fistula surgery in 6 months
Inadequate documentation of insertion date	Updating of policies and requirement for dating of PVCs	Suboptimal techniques when accessing lines especially inadequate hub disinfection	Development of maintenance policy and documentation followed by intensive manikin-based training for nurses	Temporary dialysis catheters kept for up to 60 days or more	A tunneled dialysis catheter needed to be done within 3 weeks of CKD5 diagnosis
PVCs inserted and kept for prolonged periods “just in case”	PVCs inserted only when clinically required and had to be changed after 72 hours unless a clinical risk assessment was done and documented in patient notes	CVC left in situ unnecessarily despite no clinical need	Mandatory daily CVC review and removal	High MRSA prevalence in dialysis population	Monthly MRSA screening and decolonization of all renal dialysis patients
Lack of a standard routine PVC assessment meaning that early signs of thrombophlebitis were missed	Introduction of the Visual Infusion Phlebitis (VIP) score monitoring on all PVCs.	Inappropriate line management when patients transferred from ICU to general wards with CVC	Follow-up by ICN of every patient sent to general wards with a CVC.	Suboptimal line access techniques	Updating of policies and training of all professionals accessing dialysis catheters

### Intravenous device interventions

The corrective actions included a mixture of policy development/updating, education and training, positive and negative motivators as well as—essentially—system change. In renal dialysis, targets were established to reduce the lag period from the start of dialysis until arterio-venous fistula surgery. NTDLHCs were only allowed as an emergency interim measure for no longer than 3 weeks. Within this period, and in all elective cases, a cuffed tunneled line needed to be inserted for hemodialysis.^13^ Whereas 30.1% of patients on hemodialysis had a NTDLHC in March 2011, this reduced to 0.5% in March 2017, despite a 64% increase in the hemodialysis population during the same period. This major change was only possible following the engagement of two specialist radiologists with experience in such insertions. Maintenance of dialysis lines was also improved through adoption of dressings with higher moisture vapor transmission rates (MVTR) and training of nurses in care bundles.

The Visual Infusion Phlebitis (VIP) score became a required mandatory daily assessment for each patient with a PVC.^14^ This policy modification was only possible after another system change: the introduction of PVC dressings with transparent visibility windows. The implementation of a 72-hour cutoff for PVC duration was more contentious, since publications at the time had shed doubt on its effectiveness.^15^ Nevertheless, it was determined that the level of PVC monitoring and care in the centers where the studies were held, had not yet been achieved in MDH; therefore the literature could not necessarily be transposed to the hospital situation. CVC initiatives mirrored the recommendations of the Institute of Health Improvement and were based heavily on the work of Berenholtz *et al*.^16^ They were however modified to reflect local culture. In particular, ICU nurses raised objections to supervising intensivists during insertion and, even more, to stopping the procedure if they noted any deviations from operating procedures; neither was adopted. In addition, the checklists were used more as an *aide memoire* than the more stringent application in the reference publication.

### Universal MRSA admission screening

The third key intervention was introduced in 2014 with the commencement of universal MRSA admission screening for practically all patients admitted to the hospital, other than in pediatrics and obstetrics.^17^ This intervention was also not without some controversy since consensus at the time advocated primarily risk-based MRSA screening strategies.^18^ Nevertheless, as we have already described previously,^17^ it was felt that the hyper-prevalence of MRSA colonization in admitted patients at the time (exceeding 13%) as well as local issues which made consistent risk assessment at ward level improbable, were sufficient reasons to trial a universal strategy. This decision was vindicated by a significant reduction in all MRSA infections (including HA-MRSA-B) at very reasonable annual cost of €1058 per QALY gain per year.^17^ Once again a centralized process was adopted, managed completely by care assistants employed within the MDH IPC team. They identify new daily admissions from the hospital’s patient administration system, visit the wards to perform the screening themselves directly onto culture media, and then coordinate the decolonization of all positive cases by a 5-day regimen of nasal mupirocin and chlorhexidine bathing.^19^

In order to assess the impact of the interventions on HA-MRSA-B rates, we used multiple change-point analysis using the R package changepoint (R Studio Ver 2023.03.0, United States) to detect significant shifts in the mean of the data, without prior setting of any intervention points in the time series model.^20^ Due to the retrospective nature of the data, the offline type was utilized. We adopted the binary segmentation technique proposed by Scott and Knott and avoided overestimation of the number of change points by applying penalties utilizing the Schwarz information criterion (SIC), Bayesian information criterion (BIC), Akaike information criterion (AIC) and Hannan–Quinn information criterion (HQIC).^21^ A one-tailed Mann–Whitney test was used to determine whether overall incidence in the segments following each change point was significantly different from that in the segment preceding it. To justify the use of the Mann–Whitney test, we ensured that the data within segments were stationary and independent using the Dickey–Fuller test and Ljung–Box test, respectively. A Shapiro–Wilk test was also conducted to check for normality within each segment. The non-parametric Mann–Whitney test was chosen because the normality assumption was violated on more than one occasion. R Studio was also used to conduct this analysis.

## Results

**Figure 1.**
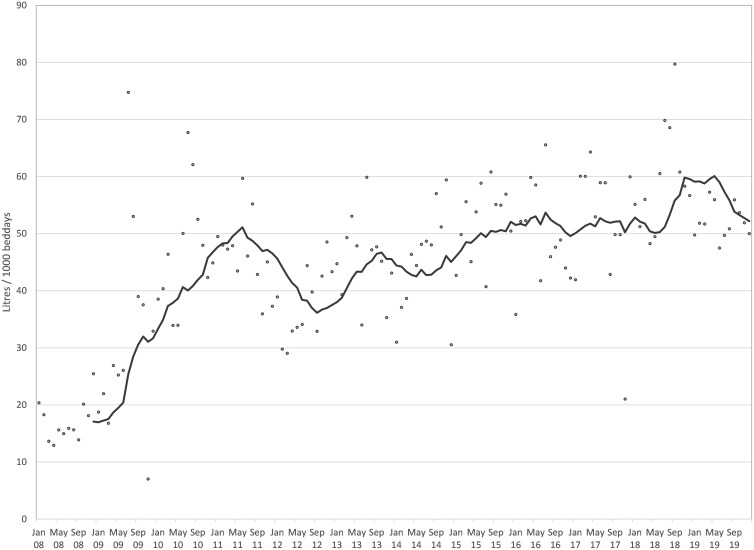
Monthly alcohol hand rub (AHR) consumption in L/1000BD (dots) with 12-month moving average (line).

**Figure 2.**
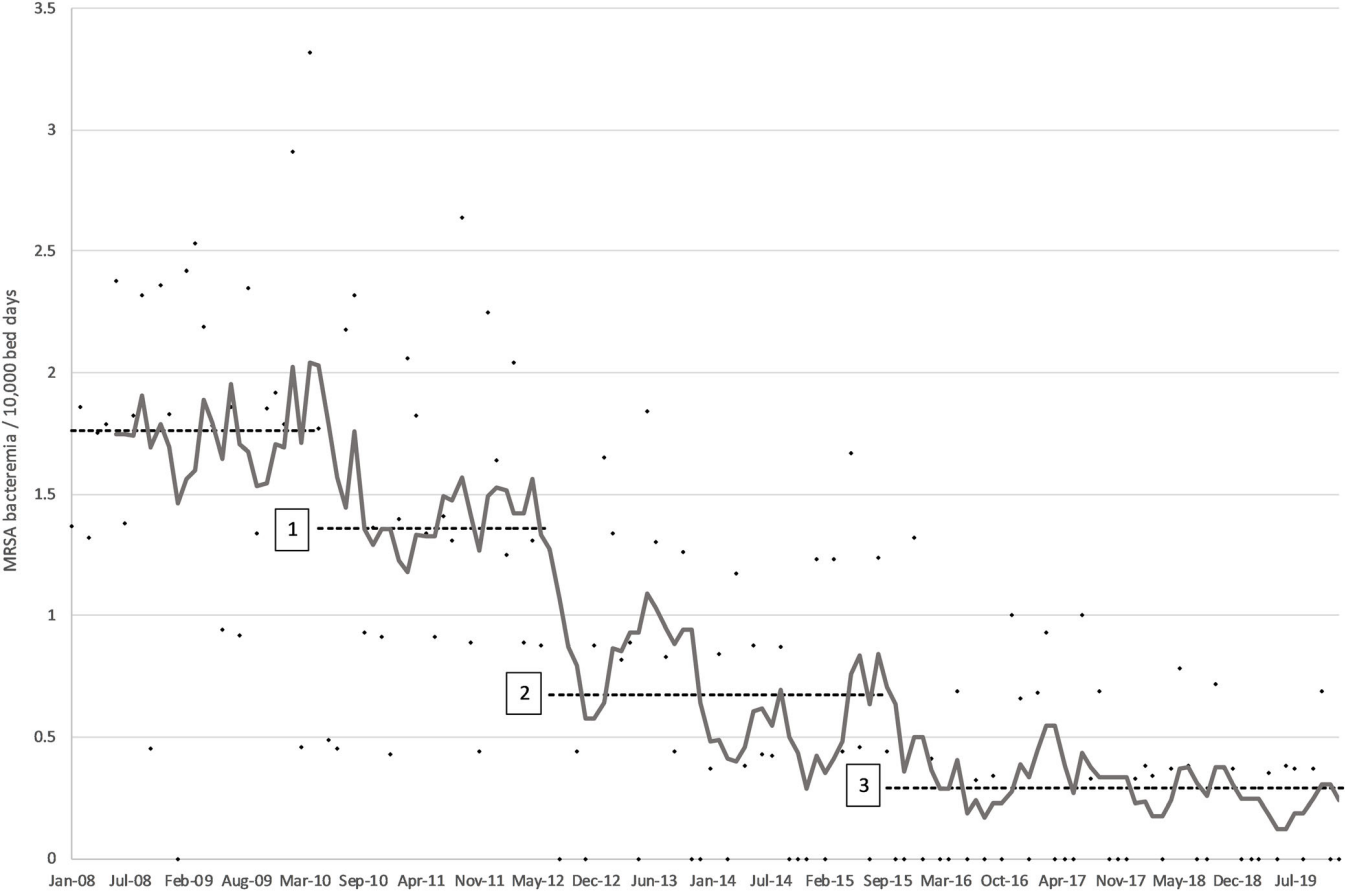
Monthly incidence of MRSA bacteremia/10000BD (dots) with 6-month moving average (gray line) and average mean incidence for baseline and each of the three change points identified (dashed line).

All four penalty variations yielded identical change points. The first change point was determined to have taken place in May 2010, with a mean improvement of 0.4 cases/10000BD from baseline (Fig. 2). This first change point coincided with reaching a monthly AHR consumption level in excess of 40 L/1000BD (Fig. 1). The second change point was reported at July 2012. It resulted in the highest average reduction of 0.688 cases/10000BD. The third change point was established in September 2015 and resulted in a further drop in incidence of 0.381 cases/10000BD. The mean yearly MRSA bacteremia rate had been greater than 1.7/10000BD before the start of the intervention (Fig. 2). In subsequent years, the rate reduced consistently—year on year—to reach 0.18/10000BD by 2019; a decrease of almost 90%. The only exception was 2015 when a spike in incidence was apparent in the first half of the year. At this time, mupirocin was not available due to supply issues and decolonization was attempted using polymyxin nasal ointment instead. The latter has been shown to be inferior to mupirocin to achieve MRSA decolonization.^22^ The Mann–Whitney test showed that, in all cases, each successive segment resulted in an overall significant decrease in incidence rate, confirming that the implied cause of the change point affected an improvement (Table 2).

**Table 2.** Mean and median incidence (cases/10,000BD) of healthcare-associated MRSA and MSSA bacteremia for each of the segments determined by change-point analysis

Change-point segment	HA MRSA bacteraemia (/10,000BD)			HA MSSA bacteraemia (/10,000BD)		
	Mean	Median	One-tailed Mann–Whitney test	Mean	Median	One-tailed Mann–Whitney test
Baseline	1.758	1.825	–	1.329	1.316	–
1. Hand hygiene campaign	1.358	1.325	*p* = 0.01	1.355	1.359	N.S.
2. IV device interventions	0.670	0.640	*p* < 0.001	0.962	0.905	*p* = 0.004
3. Universal MRSA screening	0.289	0.325	*p* < 0.001	0.992	1.035	N.S.

**Figure 3.**
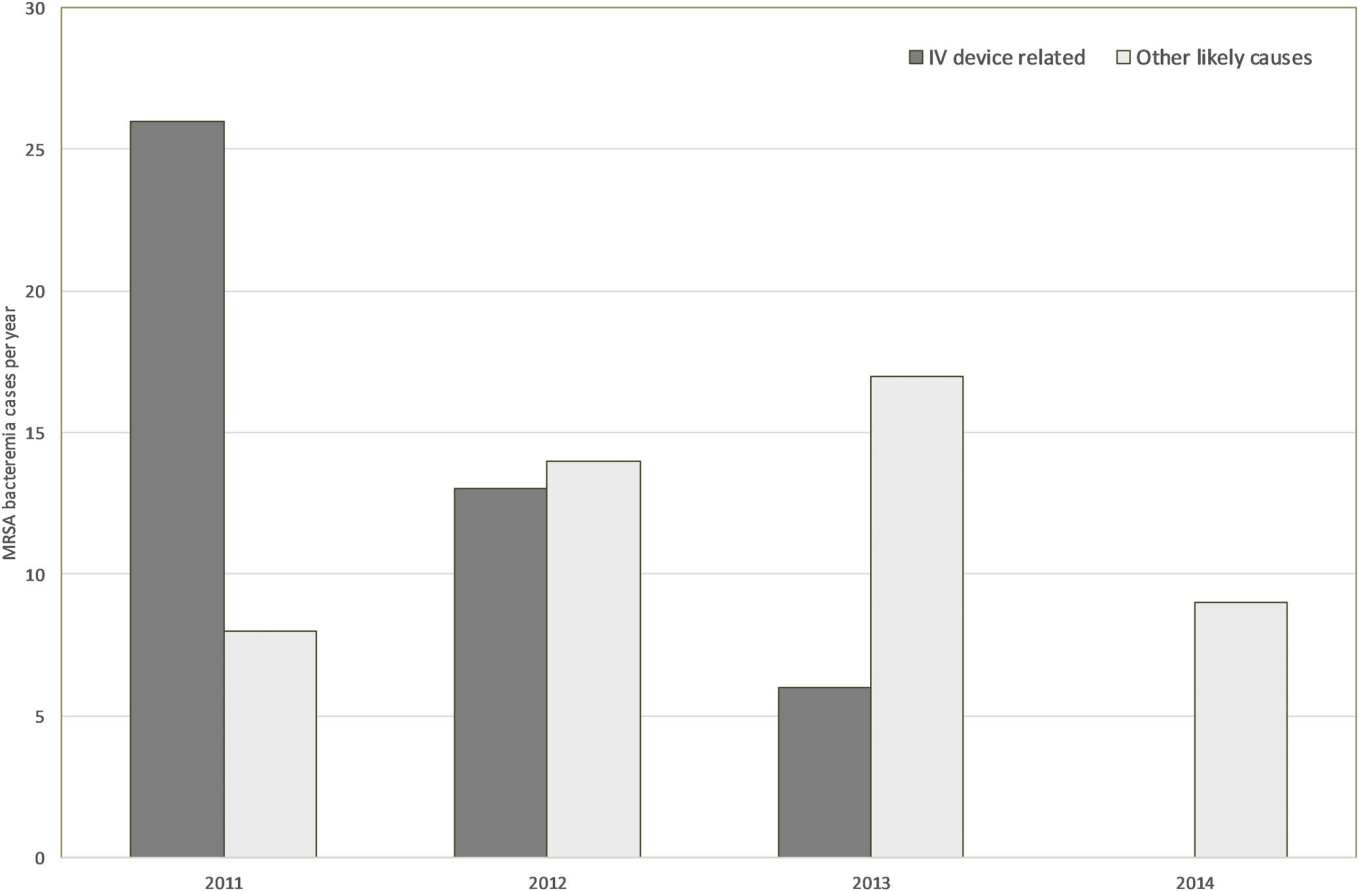
Number of MRSA bacteraemia cases (by year) after IV device interventions attributed to IV device-related factors and to other likely causes, following root cause analysis.

During 2011, 34 HA-MRSA-B cases were identified in MDH. Of these, 26 (83%) were related to intravascular devices. Following the start of corrective measures, IV-related HA-MRSA-B started to reduce: 13 in 2012 and 6 in 2013. In 2014, no such cases were identified by the RCAs. On the other hand, HA-MRSA-B related to other sources (primarily urinary, surgical site, and lung) remained relatively unchanged in number (Fig. 3). It was only when universal admission screening was introduced that the latter started to reduce as well. During the whole study period, no changes in the incidence of community-acquired MRSA bacteremia were identified. However, rates of healthcare-associated methicillin-sensitive *Staphylococcus aureus* bacteremia (HA-MSSA-B) showed a significant reduction in conjunction with the IV device interventions (Table 2). The segments relating to HH and MRSA screening initiatives did not show any differences in HA-MSSA-B.

## Discussion

The 90% overall reduction in HA-MRSA-B at MDH is arguably one of the most effective IPC interventions reported from the Mediterranean, the European region with the highest MRSA prevalence.^23^ The quasi-experimental methodology and sequential implementation allowed each intervention to be evaluated independently for effect. This is often not possible in IPC multimodal initiatives, which have often been criticized for confounding effects.^24^ Our results suggest that HH improvement, on its own, did contribute significantly toward an initial reduction in HA-MRSA-B rates. It is however to be noted that the change point happened only when AHR consumption reached 40L/1000BD, two years after the beginning of the campaign. The most recent point prevalence survey (PPS) of healthcare-associated infections (HAI) in European acute care hospitals reported that only 6 out of the 34 participating EU/EEA countries achieved this level of AHR consumption.^25^ The RCA initiative, and the corrective actions implemented as a result, had the most significant influence on HA-MRSA-B rates. RCAs have already been shown to be very effective to reduce HA-BSIs.^26–28^ However, they are often implemented as part of a bundle of measures and the specific contribution of the RCA component may be difficult to extrapolate. Our results suggest that, even on its own, RCA was a valuable tool for IPC professionals to identify possible corrective actions against HA-MRSA-B and contribute to system-wide learning within the organization.^29^ Interestingly, the IV device interventions addressing HA-MRSA-B also had a horizontal effect on HA-MSSA-B. This is not surprising since both are essentially the same organism with identical modes of transmission. On the other hand, neither the HH campaign nor MRSA admission screening impacted on HA-MSSA-B rates. Again, this is to be expected since neither are likely to impact significantly on MSSA colonization and subsequently infection.^30^

In retrospect, we believe that the initiative was successful because it employed most of the key requirements of a proper behavior change strategy, as highlighted by Kotter’s 8-step model.^31^ EU surveillance reports were utilized to instill urgency. The IPC team provided the vision, guidance, and communication to enable action. Short-term wins were celebrated while barriers addressed and overcome. Above all, transformational change was achieved by recognizing and understanding organizational and national cultural backgrounds and adapting interventions accordingly.^32,33^ This was manifest in the predominantly top-down approach, coupled with regular communication and feedback with front-line staff. It was in line with the high uncertainty avoidance and power distance that characterizes Maltese national culture.^32^ However, this heavily centralized approach and extensive day-to-day direct involvement in all aspects of the interventions placed a heavy workload on the IPC team. Procedures that were part of previously published successful studies (such as nurse supervision of CVC insertion) were modified because they were deemed to be culturally incompatible and could have undermined the whole project. Similarly, whereas evidence-based literature was used to inform the interventions, the setting where these studies were undertaken was always considered. This was seen in the decision to set a 72-hour cutoff for duration of PVCs, although this has now been replicated in more recent studies showing a lower risk for BSI following routine replacement of PVCs than based on clinical indication.^34^ We posited that the organizational context and environment of any successful study needed to be comparable to our own before the intervention was introduced.^35^ Above all, a considerable lag period (in excess of 18 months) was needed before the impact of each intervention became apparent in terms of infection outcome. This is not surprising because IPC interventions are essentially aimed at achieving behavior change. Even when eventually successful, this change needs time to materialize. Yet many IPC studies, including randomized control trials, are often organized for relatively short durations, even as little as 12 months.^36^ It should be no surprise if sufficient behavior change, that impacts on infection outcomes, cannot be achieved within such a short time interval.

In conclusion, our experience confirms that a significant reduction of multidrug-resistant HAIs is achievable, even in very high endemic settings. This can be accomplished through behavior change strategies that incorporate smart, relatively cost-neutral, interventions—such as RCAs—which provide information required for action. It is critical that interventions formulated from such data take into account, and are compatible with, local realities and culture. Lastly, because they address human behavior, a lag period is to be expected before improvement becomes evident and the implementation period needs to be long enough to take this into account.

## Data Availability

The data that support the findings of this study are available on request from the corresponding author, MAB.

## References

[1] van Hal SJ , Jensen SO , Vaska VL , Espedido BA , Paterson DL , Gosbell IB. Predictors of mortality in *Staphylococcus aureus* bacteremia. Clin Microbiol Rev 2012;25:362–386. 22491776 10.1128/CMR.05022-11 PMC3346297

[2] Blot SI , Vandewoude KH , Hoste EA , Colardyn FA. Outcome and attributable mortality in critically Ill patients with bacteremia involving methicillin-susceptible and methicillin-resistant *Staphylococcus aureus* . Arch Intern Med 2002;162: 2229–2235. 12390067 10.1001/archinte.162.19.2229

[3] Cassini A , Högberg LD , Plachouras D et al. Attributable deaths and disability-adjusted life-years caused by infections with antibiotic-resistant bacteria in the EU and the European Economic Area in 2015: a population-level modelling analysis. Lancet Infect Dis 2019;19:56–66. 30409683 10.1016/S1473-3099(18)30605-4 PMC6300481

[4] de Kraker ME , Davey PG , Grundmann H. BURDEN study group. Mortality and hospital stay associated with resistant *Staphylococcus aureus* and *Escherichia coli* bacteremia: estimating the burden of antibiotic resistance in Europe. PLoS Med 2011;8:e1001104. 22022233 10.1371/journal.pmed.1001104 PMC3191157

[5] European Centre for Disease Prevention and Control. Antimicrobial resistance surveillance in Europe 2015. Annual Report of the European Antimicrobial Resistance Surveillance Network (EARS-Net). Stockholm: ECDC; 2017.

[6] Davey P , Sneddon J , Nathwani D. Overview of strategies for overcoming the challenge of antimicrobial resistance. Expert Rev Clin Pharmacol 2010;3:667–686, 22111749 10.1586/ecp.10.46

[7] Pittet D , Allegranzi B , Storr J , Donaldson L. ‘Clean care is safer care': the global patient safety challenge 2005–2006. Int J Infect Dis 2006;10:419–424. 16914344 10.1016/j.ijid.2006.06.001

[8] Abela N , Borg MA. Impact on hand hygiene compliance following migration to a new hospital with improved resources and the sequential introduction of World Health Organization recommendations. Am J Infect Control 2012;40:737–741. 22285712 10.1016/j.ajic.2011.09.012

[9] Borg MA , Brincat A. Addressing the controversy of 100% hand hygiene compliance: can alcohol rub consumption data serve as a useful proxy validator? J Hosp Infect 2018;100:218–219. 29733923 10.1016/j.jhin.2018.04.024

[10] Duerden B , Fry C , Johnson AP , Wilcox MH. The control of Methicillin-resistant *Staphylococcus aureus* blood stream infections in England. Open Forum Infect Dis 2015;2:ofv035. 26380336 10.1093/ofid/ofv035 PMC4567090

[11] Department of Health. Going Further Faster II: Applying the Learning to Reduce HCAI and Improve Cleanliness. London: COI; 2008.

[12] Venier AG. Root cause analysis to support infection control in healthcare premises. J Hosp Infect 2015;89:331–334. 25634490 10.1016/j.jhin.2014.12.003

[13] Gallieni M , Brenna I , Brunini F , Mezzina N , Pasho S , Giordano A. Dialysis central venous catheter types and performance. J Vasc Access 2014;15:S140–S146. 24817472 10.5301/jva.5000262

[14] Morris W , Heong Tay M. Strategies for preventing peripheral intravenous cannula infection. Br J Nurs 2008;17:S14–S21. 10.12968/bjon.2008.17.Sup8.31470 18974681

[15] Rickard CM , McCann D , Munnings J , McGrail MR. Routine resite of peripheral intravenous devices every 3 days did not reduce complications compared with clinically indicated resite: a randomised controlled trial. BMC Med 2010;8:53. 20831782 10.1186/1741-7015-8-53 PMC2944158

[16] Berenholtz SM , Pronovost PJ , Lipsett PA et al. Eliminating catheter-related bloodstream infections in the intensive care unit. Crit Care Med 2004;32:2014–2020. 15483409 10.1097/01.ccm.0000142399.70913.2f

[17] Borg MA , Suda D , Scicluna E , Brincat A , Zarb P Universal admission screening: a potential game-changer in hospitals with high prevalence of MRSA. J Hosp Infect 2021;113:77–84. 33811962 10.1016/j.jhin.2021.03.024

[18] Robotham JV , Deeny SR , Fuller C , Hopkins S , Cookson B , Stone S. Cost-effectiveness of national mandatory screening of all admissions to English National Health Service hospitals for meticillin-resistant *Staphylococcus aureus*: a mathematical modelling study. Lancet Infect Dis 2016;16:348e56. 26616206 10.1016/S1473-3099(15)00417-X

[19] Septimus E , Weinstein RA , Perl TM , Goldmann DA , Yokoe DS. Approaches for preventing healthcare-associated infections: go long or go wide? Infect Control Hosp Epidemiol 2014;35:797–798. 24915206 10.1086/676535

[20] Killick, R , Eckley, IA. Change-point: an R package for change-point analysis. J Stat Softw 2014;58:1–19.

[21] Scott AJ , Knott M. A cluster analysis method for grouping means in the analysis of variance. Biometrice 1974;30:507–512.

[22] O’Grady S , Hirji Z , Pejcic-Karapetrovic B , et al. A double-blind, randomized, controlled trial of topical polysporin triple compound versus topical mupirocin for the eradication of colonization with methicillin-resistant *Staphylococcus aureus* in a complex continuing care population. Can J Infect Dis Med Microbiol 2009;20:e49–e55. 20808456 10.1155/2009/274896 PMC2770302

[23] Borg MA , Camilleri L. What is driving the epidemiology of Methicillin-resistant *Staphylococcus aureus* infections in Europe? Microb Drug Resist 2021;27:889–894. 33337277 10.1089/mdr.2020.0259

[24] McLaws ML. The relationship between hand hygiene and health care-associated infection: it’s complicated. Infect Drug Resist 2015;8:7–18. 25678805 10.2147/IDR.S62704 PMC4319644

[25] European Centre for Disease Prevention and Control. Point Prevalence Survey of Healthcare-Associated Infections and Antimicrobial Use in European Acute Care Hospitals, 2016–2017. Stockholm: ECDC; 2023.

[26] Hallam C , Jackson T , Rajgopal A , Russell B. Establishing catheter-related bloodstream infection surveillance to drive improvement. J Infect Prev 2018;19:160–166. 30013620 10.1177/1757177418767759 PMC6039910

[27] Török ME , Harris SR , Cartwright EJ , et al. Zero tolerance for healthcare-associated MRSA bacteraemia: is it realistic? J Antimicrob Chemother 2014;69:2238–2345. 24788657 10.1093/jac/dku128 PMC4100711

[28] Sood G , Caffrey J , Krout K , et al. Use of implementation science for a sustained reduction of central-line-associated bloodstream infections in a high-volume, regional burn unit. Infect Control Hosp Epidemiol 2017;38:1306–1311. 28899444 10.1017/ice.2017.191

[29] Taitz J , Genn K , Brooks V , et al. System-wide learning from root cause analysis: a report from the New South Wales Root Cause analysis review committee. Qual Saf Health Care 2010;19:e63. 20671073 10.1136/qshc.2008.032144

[30] Nurjadi D , Eichel VM , Tabatabai P , et al. Surveillance for colonization, transmission, and infection with methicillin-susceptible *Staphylococcus aureus* in a Neonatal Intensive Care Unit. JAMA Netw Open 2021;4:e2124938. 34515783 10.1001/jamanetworkopen.2021.24938 PMC8438598

[31] Campbell RJ. Change management in health care. Health Care Manag 2020;39:50–65. 10.1097/HCM.0000000000000290 32345939

[32] Borg MA . Lowbury Lecture 2013. Cultural determinants of infection control behaviour: understanding drivers and implementing effective change. J Hosp Infect 2014;86:161–168. 24534705 10.1016/j.jhin.2013.12.006

[33] De Bono S , Heling G , Borg MA. Organizational culture and its implications for infection prevention and control in healthcare institutions. J Hosp Infect 2014;86:1–6. 24309419 10.1016/j.jhin.2013.10.007

[34] Buetti N , Abbas M , Pittet D , et al. Comparison of routine replacement with clinically indicated replacement of peripheral intravenous catheters. JAMA Intern Med 2021;181:1471–1478. 34533191 10.1001/jamainternmed.2021.5345 PMC8561330

[35] Dixon-Woods M , Leslie M , Tarrant C , Bion J. Explaining matching Michigan: an ethnographic study of a patient safety program. Implement Sci 2013;8:70. 23786847 10.1186/1748-5908-8-70 PMC3704826

[36] Teesing GR , Richardus JH , Nieboer D , et al. The effect of a hand hygiene intervention on infections in residents of nursing homes: a cluster randomized controlled trial. Antimicrob Resist Infect Control 2021;10:80. 34016156 10.1186/s13756-021-00946-3 PMC8138990

